# Effects of Aerobic Exercise in the Intensive Care Unit on Patient-Reported Physical Function and Mental Health Outcomes in Severely Burned Children—A Multicenter Prospective Randomized Trial

**DOI:** 10.3390/jpm13030455

**Published:** 2023-02-28

**Authors:** Alen Palackic, Andrea Rego, Ingrid Parry, Soman Sen, Ludwik K. Branski, Taylor G. Hallman, Heidi Spratt, Jong O. Lee, David N. Herndon, Steven E. Wolf, Oscar E. Suman

**Affiliations:** 1 Department of Surgery, School of Medicine, University of Texas Medical Branch, Galveston, TX 77555, USA; 2 Division of Plastic, Aesthetic and Reconstructive Surgery, Department of Surgery, Medical University of Graz, 8036 Graz, Austria; 3 Department of Surgery, University of California, Davis, CA 95616, USA; 4 Department of Biostatistics and Data Science, University of Texas Medical Branch, Galveston, TX 77555, USA; 5 Oxford University Press, Wolters Kluwer N.V., 2400BA Alphen aan den Rijn, The Netherlands

**Keywords:** burns, rehabilitation, exercising, intensive care unit

## Abstract

Severe burns are life-altering and can have lasting effects on patients’ physical and mental health. Alterations in physical function, changes in appearance, and psychological disturbances resulting from severe burns are especially concerning in children, as they are still in the early stages of identity formation. Exercise in the nonburn population has been shown to improve quality of life and result in better physical and mental status. However, the effect of early exercise on the quality of life in pediatric burn patients requires more research. Methods: Forty-eight children between the ages of seven and seventeen with ≥30% total body surface area (TBSA) burn were randomized in a 1:2 fashion to receive treatment with standard-of-care (SOC) or standard-of-care plus exercise (SOC+Ex). Surveys administered at admission and discharge collected patient-reported information regarding physical and mental health outcomes. The results are given as means +/− standard deviation. Significance was set at *p* < 0.05. Results: The average age of the SOC and SOC+Ex groups were 12 ± 3 and 13 ± 4 years, respectively. The average %TBSA burned in the SOC and SOC+Ex groups were 54 ± 17 and 48 ± 14, respectively. The SOC+Ex group averaged 10 ± 9 exercise sessions (range of 1 to 38 sessions) with an attendance rate of 25% (10 sessions out of 40 BICU days). Both groups demonstrated significant improvement in patient-reported physical and mental outcomes during hospital admission (*p* < 0.05) However, additional exercise did not exhibit any additional benefits for measured levels. Conclusions: Our recommendation is for all pediatric patients in the BICU to continue with the SOC and consult with their physician over the benefits of additional aerobic exercise. This study suggests that perhaps there is potential for increasing the amount of exercise that can be administered to pediatric burn survivors beyond SOC as we did not find aerobic exercise to be of any harm to any patients if it is performed properly and under supervision.

## 1. Introduction

Severe burns can have devastating physical and mental consequences. Permanent physical disfiguration is a common sequela of severe burns, resulting from both the burn itself and the numerous surgical procedures used to treat the burn [1]. Functional deficits can also arise following severe burn due to pain and/or reduced mobility from scar contractures [2]. Lastly, psychological trauma can develop from the burn event and challenges during recovery [2,3]. These consequences inflict a significant burden on burn survivors that can prove to be detrimental to their quality of life. Studies have shown that burn patients experience reduced physical functioning and high levels of psychological distress, anxiety, depression, and loss of behavioral and emotional control relative to non-burn normative values [4].

Physical exercise has many well-known benefits; it is associated with decreased cardiovascular disease, improved joint health, and increased energy levels [5]. Exercise has also been shown to positively impact mental health, with studies reporting improved sleep and mood [6,7]. These beneficial effects have been demonstrated when exercise has been incorporated into an outpatient treatment regimen [8]. However, the initiation to exercise in these patients is often postponed until the patient is discharged from the hospital. Due to the fact that hospitalizations related to severe burn injuries are often lengthy, a delay in exercise initiation allows for the development of significant muscle atrophy and subsequent reduced capacity for physical function. Prior studies have shown that there is currently no well-defined standard-of-care in regard to starting exercise programs in the ICU. However, the beneficial effects of exercise when started early in the ICU have been reported [9,10]. The true effects of continuing exercise after discharge from the ICU are less well known, as is the case for whether induced benefits seen in the ICU are equivalent to benefits post-discharge due to limited studies and available data on the topic [11]. A small study in adult patients discharged from the ICU displayed high adherence and acceptance to exercise interventions, making these interventions more feasible [12]. A different study used a 12-week exercise regimen on pediatric patients recently discharged from the ICU and found that implementing such program may be beneficial in promoting physical and psychosocial outcomes [13].

Therefore, we sought to evaluate the effects of an aerobic exercise program administered to pediatric burn patients while in the burn intensive care unit (BICU) on self-reported physical and mental health outcomes. Our hypothesis is that the addition of aerobic exercise to standard-of-care (SOC) treatment while in the BICU will be superior to SOC treatment alone in improving patient-reported physical and mental health outcomes at hospital discharge.

## 2. Materials and Methods

### 2.1. Subjects

Children 7–17 years of age admitted to the BICU at the Shriners Hospital for Children in Galveston, TX or Shriners Hospital for Children in Sacramento, CA between 2014 and 2018 were prospectively considered for enrollment in this study. Patients were not eligible for participation in the trial if the etiology of their burns was purely electrical; if they had a diagnosis of tuberculosis; or if they had severe behavioral and/or cognitive disorders (e.g., intellectual disability, autism) that would prevent safe participation in an exercise program. Patients were eligible for enrollment in the study if their total body surface area (TBSA) burned was greater-than-or-equal-to 30% and they did not meet any of the criteria for disqualification. Parents provided signed informed consent for all patients. Children 12 years and older provided signed informed consent in addition to the parents’ consent. Finally, if the patient was 7–12 years old, he/she provided assent in place of consent.

### 2.2. Study Design

Enrolled participants were randomized in a 2:1 fashion to receive SOC+Ex or SOC and were subsequently administered surveys assessing physical and mental health outcomes at admission and discharge. The randomization process was performed by a senior clinical biostatistician at UTMB and was designed to increase the likelihood of potential benefit to patients. This method, also described in our group’s previous publication on physical exercise outcomes, allowed for the ability to extend randomization and enrollment numbers to account for mortality and dropouts as needed. 

SOC measures included a range of motion activities, functional mobility and gait exercises, scar management, and patient/family training. Patients began their respective treatment regimens only after it was deemed safe for them to do so. All study participants were monitored for safety and for the development of any contraindications to participating in the study.

### 2.3. Aerobic Exercise (Ex) Intervention

All children with severe burns were treated with the individualized SOC at the Shriners Hospitals for Children in Galveston, TX and Sacramento, CA. Due to the fact that this study was designed to investigate the efficacy of SOC+Ex, there were no limitations on activities, treatment, or medications that were considered part of SOC.

The aerobic exercise intervention involved measurable workloads using leg and arm cycle ergometers (Monark Rehab Trainer 881-E). Treatment sessions were individualized such that each participant performed the exercises at a moderate-to-hard effort as measured by a rated perceived exertion (RPE) scale; this scale ranged from 1 to 10, where “3” indicated moderate effort, and “5” indicated hard effort [14].

To determine the optimal exercise load for each patient, an introductory exercise session was completed. This introductory session consisted of an initial 5-watt exercise load and recording of RPE after one minute, followed by 5-watt increases until an RPE of 3 and 5 were reached for respiratory and muscle effort. The exercise load at which these values were achieved would serve as the baseline for all future training sessions.

Exercise sessions occurred twice daily from Monday to Friday, one morning session for the lower extremities (LEs) and one afternoon session for the upper extremities (UEs). No exercise sessions were conducted on Saturdays and Sundays. Exercise training continued daily until the subject was discharged from the BICU. Measurements for both the SOC and SOC+Ex groups were assessed immediately before and after one short bout of exercise was completed. This was performed early during the BICU stay and also performed at discharge from the BICU. Thus, there were two exercise bouts (one early in BICU stay and one at discharge from the BICU), with pre and post single short bout measurements The initial bout for both groups was the first permissible session of aerobic exercise. The final bout was conducted at discharge. A detailed description of the exercise regimen is published elsewhere [15].

### 2.4. Survey

Surveys administered at admission and discharge were used to collect information on patient-reported physical and mental health outcomes (see Supplementary Materials Figure S1). We developed these surveys internally, using a combination of our authors’ clinical experience and a review of published literature to inform its content. The survey was constructed based upon the opinions of burn therapists as well as the common concerns or complaints of patients and their families, in which pain, weakness, tiredness, sleep, sadness, and misery were some of the more commonly reported. The survey aimed to achieve a consensus of opinion on a topic where there is little or no definitive evidence other than expert opinion. The self-reported physical outcomes assessed included pain, weakness, and tiredness. Each outcome was measured on a scale of 0 to 4, with 0 indicating no pain, weakness, or tiredness and 4 a significant amount. The mental health outcomes were measured by sleep, sad, and misery scores and also measured on a scale from 0 to 4 for each outcome. Additionally, self-reported physical outcomes at baseline and at discharge for a single short bout of exercise were also recorded on a scale of 0–4. Here, baseline is considered the first permissible session of aerobic exercise. The primary aim was to measure the effect of additional aerobic exercise training on physical and mental health status from admission to discharge. The secondary aim was to measure the effect of a single short bout of exercise on physical outcomes. For the initial short bout of exercise and the final short bout of exercise, mental health outcomes were not measured.

### 2.5. Statistical Analysis

Analysis was performed after controlling for age, gender, length of stay, number of exercise sessions, and %TBSA. Two-way repeated measures ANOVA was used to assess whether an outcome measure had a statistically significant change from admission to discharge. To obtain information on associations between an outcome level with treatment type, length of stay, %TBSA, and number of exercise treatments, a linear regression model was used. Results are stated as mean ± SD; significance was set at *p* < 0.05. As limited data exist on self-reported scores for physical and mental health in this special patient population, we were unable to estimate precision around the variables we planned to collect.

## 3. Results

### 3.1. Demographics and Clinical Characteristics of the Study Population

**Figure 1:**
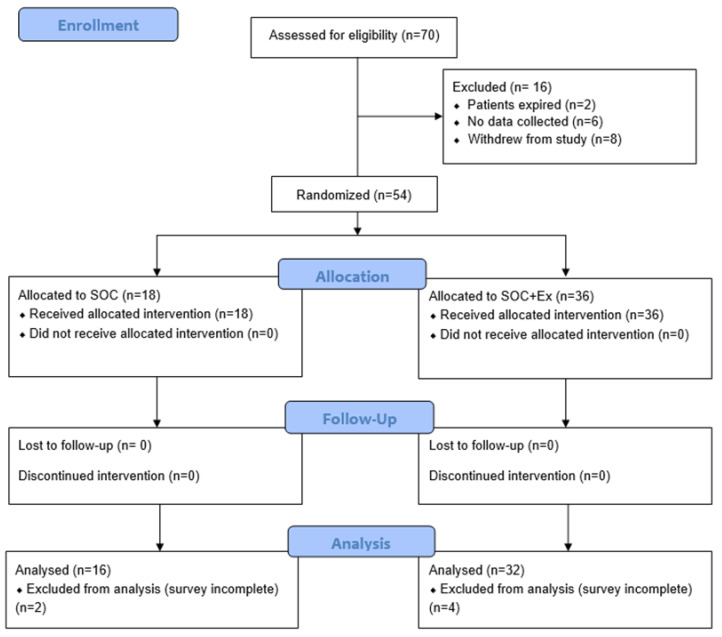
Flowchart illustrating study participant assessment for eligibility, randomization, enrollment, allocation to treatment group, follow-up, and analysis. SOC, standard of care; SOC+Ex, standard of care plus exercise.

Of the 70 participants assessed for eligibility, 54 participants were eligible and randomized to treatment groups. Participants were allocated to the SOC group (n = 18, 68.75% male children) and the SOC+Ex group (n = 36, 90.6% male children). A total of 48 participants were included into the final analysis, because 6 participants could not complete the surveys. Figure 1 shows a flow diagram illustrating the recruitment and retention process. The average age of the SOC and SOC+Ex groups were 12 ± 3 and 13 ± 4 years, respectively. The average %TBSA burned in the SOC and SOC+Ex groups were 54 ± 17 and 48 ± 14 and TBSA third-degree burns 43 ± 24 and 36 ± 20, respectively. Both groups were comparable in age and severity of the burn injury. The median number of length of stay (LOS) in the BICU for the SOC group was 51 ± 23 days (ranging from 21 to 143 days), while the SOC+Ex group had an average LOS of 40 ± 19 days, ranging from 18 to 97 days. Despite large ranges, the groups were comparable in LOS. The SOC+Ex group averaged 10 ± 9 exercise sessions (range of 1 to 38 session) with an attendance rate of 25%. Participants started exercising 23 ± 13 days after admission (Figure 1).

### 3.2. Self-Reported Physical Outcomes between Admission and Discharge Prior to the Single Short Bout of Exercise

**Figure 2:**
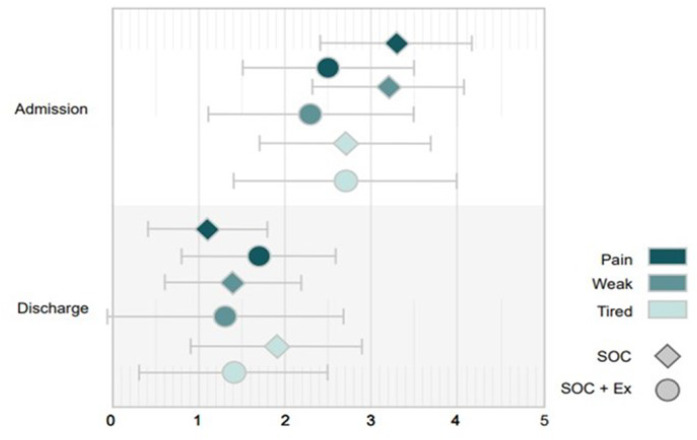
Patient-reported physical item scores at admission and discharge. Different coloring indicates physical score items: pain, weak, tired. All scores were lower at discharge than at admission, indicating improvement (*p* < 0.05). Adding exercise had no additional benefits. SOC, standard of care; SOC+Ex, standard of care plus exercise.

The average pain scores for the SOC and SOC+Ex group were 3.3 ± 0.9 and 2.5 ± 1 at baseline and 1.1 ± 0.7 and 1.7 ± 0.9 at discharge, respectively. The average weakness scores for the SOC and SOC+Ex group were 3.2 ± 0.9 and 2.3 ± 1.2 at baseline and 1.4 ± 0.8 and 1.3 ± 1.4 at discharge, respectively. The average tiredness scores for the SOC and SOC+Ex group were 2.7 ± 1.0 and 2.7 ± 1.3 at baseline and 1.9 ± 1.0 and 1.4 ± 1.1 at discharge, respectively. The discharge timepoint was significantly decreased from the admission timepoint in terms of the pain, weakness, and tiredness scores in both groups (*p* < 0.05). (Figure 2) There was also a significant interaction between timepoint and treatment for the pain score (*p* < 0.05). There was a significant difference between treatment types for the weakness score (*p* < 0.05).

When controlling for age, gender, length of stay, number of exercise sessions, and %TBSA, treatment was not significant between groups when examining one’s pain (*p* = 0.4505), weakness (*p* = 0.3457), and tiredness (*p*-value = 0.9651) levels. However, when controlling for age, gender, length of stay, number of exercise sessions, and %TBSA, timepoint makes a difference in pain, weakness, and tiredness levels (*p* < 0.0001). There was also a significant interaction between timepoint and treatment for the pain (*p* = 0.0054) and weakness (*p* = 0.0034) scores. For tiredness, there was not a significant interaction (*p* = 0.2121) between timepoint and treatment. Linear regression modelling revealed that there was no difference in pain, weakness, and tiredness levels due to treatment at any timepoint.

### 3.3. Self-Reported Mental Health Outcomes between Admission and Discharge Prior to the Single Short Bout of Exercise

**Figure 3:**
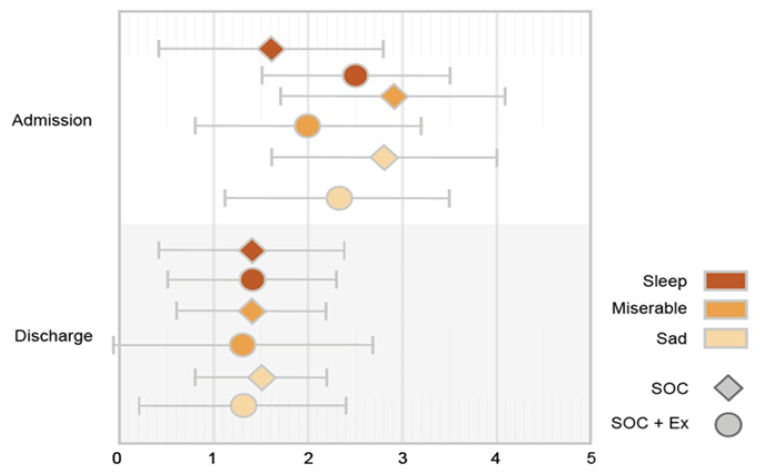
Patient-reported mental item scores at admission and at discharge. Different coloring indicates mental score items: pain, weak, tired. All scores were lower at discharge than admission, indicating improvement (*p* < 0.05). Adding exercise had no additional benefits. SOC, standard of care; SOC+Ex, standard of care plus exercise.

**Figure 4:**
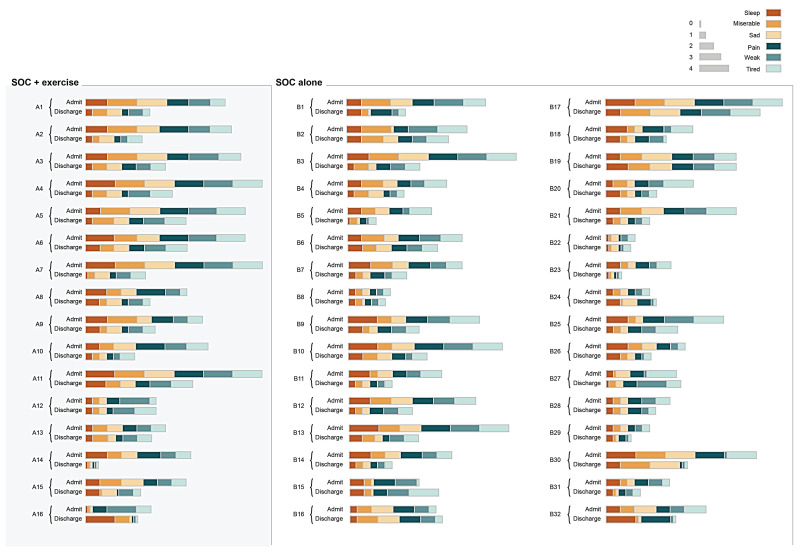
Heatmap illustrating patient-reported physical and mental item scores at admission and at discharge for individual study participants (within their respective groups). Different coloring indicates self-reported physical and mental health items: pain, weak, tired, sleep, misery, and sad. The width of the color corresponds to the score of each parameter (0 = minimal, 4 = maximum). SOC, standard of care; SOC+Ex, standard of care plus exercise.

The average sleep scores for the SOC and SOC+Ex group were 2.6 ± 1.2 and 2.5 ± 1 at baseline and 1.4 ± 1 and 1.4 ± 0.9 at discharge, respectively. The average sad scores for the SOC and SOC+Ex group were 2.8 ± 1.2 and 2.3 ± 1.2 at baseline and 1.5 ± 0.7 and 1.2 ± 1.1 at discharge, respectively. The average misery score for the SOC and SOC+Ex group were 2.9 ± 1.2 and 2.0 ± 1.2 at baseline and 1.4 ± 0.8 and 1.3 ± 1.1 at discharge, respectively. The discharge timepoint was significantly decreased from the admission timepoint in terms of the sleep, sad, and misery scores in both groups (*p* < 0.05) (Figure 3). There was also a significant interaction effect between time and treatment for the misery score (*p*-value = 0.029). When controlling for age, gender, length of stay, number of exercise sessions, and %TBSA, treatment was not significant when examining one’s sleep (*p* = 0.3246), sadness (*p* = 0.081), and misery (*p* = 0.084) level. However, when controlling for age, gender, length of stay, number of exercise sessions, and %TBSA, time made a difference in sleep, sadness, and misery levels (*p*-value < 0.0001). There was also a significant interaction (*p* = 0.0285) between time and treatment for the misery score. Figure 4 illustrates individual scores for all measured self-reported physical and mental outcomes at admission and discharge.

### 3.4. Self-Reported Physical Outcomes at Admission and at Discharge after the Single Short Bout of Exercise

**Figure 5:**
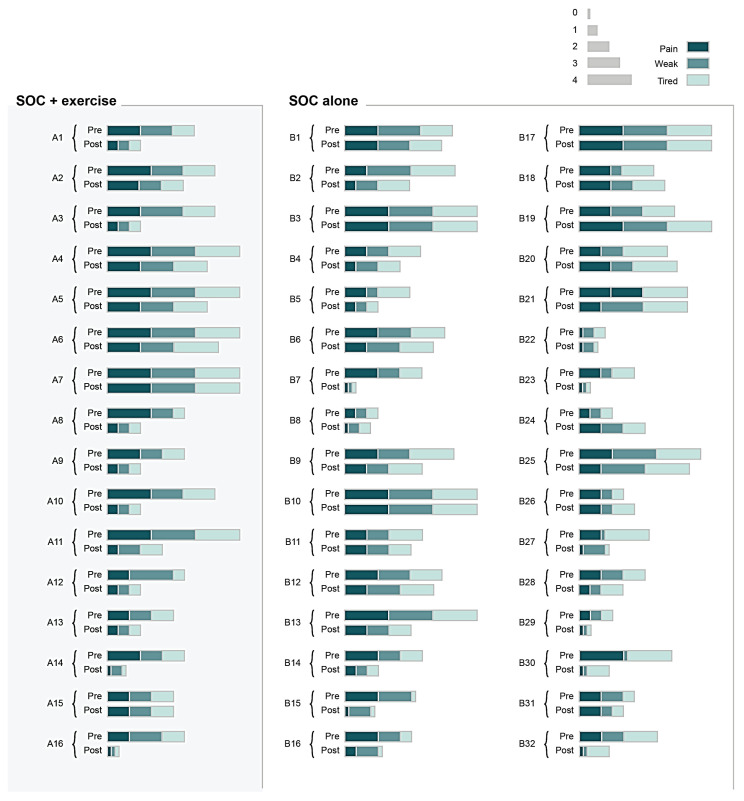
Heatmap illustrating patient-reported physical item scores at admission for individual study participants (within their respective group) before and after the single short bout. Different coloring indicates self-reported physical items: pain, weak, tired. The width of the color corresponds to the score of each parameter (0 = minimal, 4 = maximum). SOC, standard of care; SOC+Ex, standard of care plus exercise.

**Figure 6:**
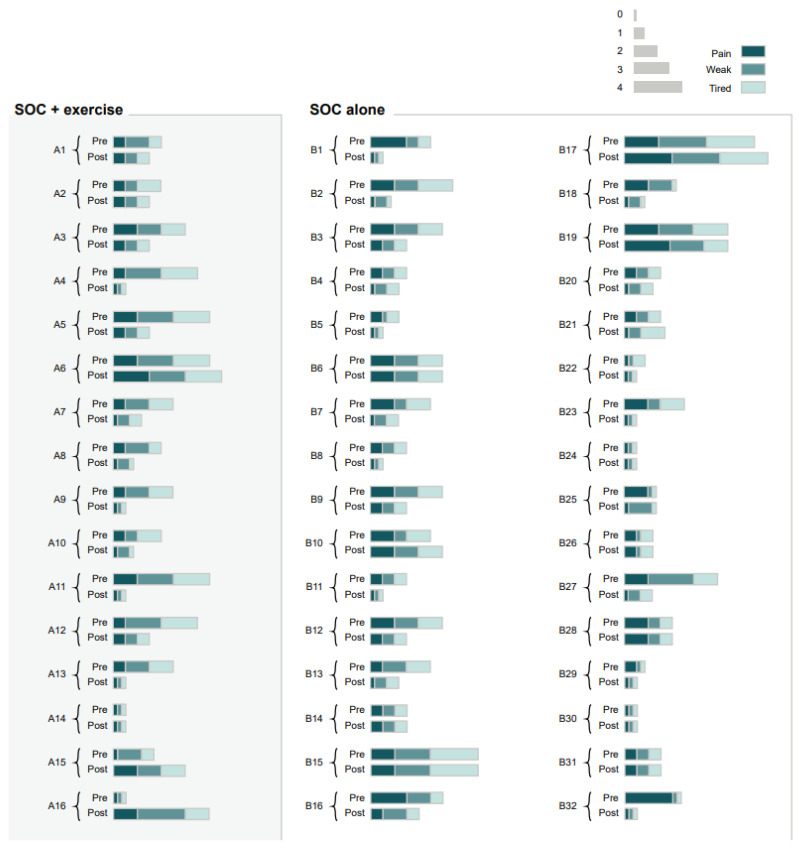
Heatmap illustrating patient-reported physical item scores at discharge for individual study participants (within their respective group) before and after the single short bout. Different coloring indicates self-reported physical items: pain, weak, tired. The width of the color corresponds to the score of each parameter (0 = minimal, 4 = maximum). SOC, standard of care; SOC+Ex, standard of care plus exercise.

At admission (baseline), scores for pain, weakness, and tiredness were significantly higher (worse) than scores reported after the single short bout of exercise (*p* < 0.05) (Figure 5). Furthermore, there was also a significant interaction effect between timepoint and treatment for the weakness score (*p* < 0.05). Similarly, scores for pain, weakness, and tiredness were significantly reduced after the single short bout of exercise at discharge. (*p* < 0.05) (Figure 6). There were no significant differences between treatment types or the interaction between timing of exercise (admission or discharge) and treatment. 

## 4. Discussion

We found that there was a significant difference for all self-reported physical outcome scores between admission and discharge in both treatment groups, but no significant difference between treatment groups. In other words, both SOC and SOC+Ex were effective in improving physical outcomes in the pediatric burn population during their BICU stay. It is important to note that, although we did not find any significant difference in physical outcomes scores between treatment groups, the course of patient-reported improvements was different. The SOC+Ex group demonstrated an improvement or lower reported levels of weakness and fatigue from admission to discharge (pre- and post-exercise). In contrast, the SOC group reported higher levels of weakness and fatigue right before a single short bout of exercise, regardless of whether it was performed at admission or discharge. However, these scores only improved (less fatigue/weakness) after (i.e., post) the single short bout of exercise conducted at discharge. This finding may indicate that the addition of an aerobic exercise program to SOC is safe and potentially beneficial, as no identifiable adverse events or harm were reported. 

Our findings regarding patient-reported mental outcomes were similar to the findings for patient-reported physical outcomes: both treatment groups exhibited significant improvement in their scores from admission to discharge, but there was no significant difference in scores between treatment groups. For the sadness and sleep measures, there was no significant interaction effect between time and treatment. However, there was a significant interaction effect between time and treatment for misery scores. Therefore, the addition of aerobic exercise significantly improved mental health outcome in conjunction with time. When controlling for age, gender, length of stay, number of exercise sessions, and %TBSA, time makes a difference in misery, sadness, and sleep scores. We note that across all outcomes, whether physical or mental, there was a significant improvement from time of admission to discharge. This comes as no surprise as the SOC should improve all of the outcomes. Although additional aerobic exercise did not improve outcomes alone, it did display significant improvement in conjunction with time for some measures. This is more in line with previous research that demonstrated the positive effects of exercise when started early and on overall health-related quality of life [9,10]. We also assessed the effects of single short bouts of exercise on patient-reported physical outcomes and found a significant decrease in pain, weakness, and fatigue after exercise at both admission and discharge. This gives us more insight into the more immediate benefits of aerobic exercise in the BICU. The improvement seen in physical outcomes after just one bout of exercise advocates for its use in rehabilitation even on a more inconsistent basis based on the patient’s needs.

This study adds to the existing literature in support of the role of aerobic exercise in burn rehabilitation. The existing literature is rather generalized and addresses broad topics such as the overall role of exercise in physical and mental outcomes. This includes the known beneficial effects of exercise when started early in the ICU [9,10,11,12,13,16,17,18,19]. This study served to add more granular information about the role of aerobic exercise in the ICU. However, because 40 out of 48 (83.33%) participants were White–Hispanic, the study’s generalizability may be limited. Similarly, our findings are limited to this special population of adolescent burn patients, as we did not have a comparable score report for the general healthy population. We add to this that the self-reported outcomes were not validated with objective measures; however, this is not uncommon in self-reported measures, which do not concurrently assess the degree of association between the self-reported survey and an objective measurement or vice versa to establish validity. However, we note that these were repeated measures in the same subjects during the BICU stay. One confounding factor of the study is unavoidable when studying adolescent participants. As participant ages in the study ranged from 7 to 17, it is likely that their understanding of the surveys was variable. This was minimized as much as possible by simplifying the surveys so that all participants could understand. However, it is still possible that some participants’ ”understanding” of the scores could have been suboptimal. Although this study utilized a multicenter approach, the size of the study was relatively small, in part due to the strict exclusion criteria. A larger study would improve the low power of this study. This study’s primary outcome focused on inpatient rehabilitation during their stay in the BICU. However, there was a large range in the number of exercise sessions that patients participated in during their BICU stay. Surgeries or infections sometimes prevented a patient from participating in exercise sessions. It is likely that the decreased number of exercise sessions in some participants of the SOC+Ex group diluted the effects of the exercise. The strength of this study was improved by its multicenter approach with participants from more than one area of the United States. Additionally, no participants were lost to follow-up. There was no significant problem with participants not completing the surveys as all completed the survey. Finally, the exclusion criteria helped ensure that the exercise intervention could be properly followed through.

## 5. Conclusions

Our recommendation is for all patients in the pediatric burn population to continue with the standard-of-care and consult with their physician about the benefits of aerobic exercise. This study suggests that perhaps there is potential for increasing the amount of exercise that can be administered to pediatric burn survivors beyond SOC as we did not find aerobic exercise to be of any harm to any patients if it is performed properly and under supervision. To have a more statistically significant and representative participant population, more multicenter studies need to be conducted. Additionally, studies should follow participants beyond their stay in the BICU to assess long-term effects and feasibility. As full rehabilitation post-burn could potentially take multiple years, looking beyond the first 2 months of recovery could prove to be beneficial.

## Data Availability

Detailed data supporting the results are available from the authors.

## Supplementary Materials

The following supporting information can be downloaded at: https://www.mdpi.com/article/10.3390/jpm13030455/s1, Figure S1: Surveys administered at admission and at discharge to collect information on patient-reported physical and mental health outcomes.
