# Supplementary material for: Effects of Aerobic Exercise in the Intensive Care Unit on Patient-Reported Physical Function and Mental Health Outcomes in Severely Burned Children—A Multicenter Prospective Randomized Trial
Source: J Pers Med. 2023 Feb 28;13(3):455. doi: 10.3390/jpm13030455 (PMC10058069; doi:10.3390/jpm13030455)
Supplement: Supplementary file 1 [file jpm-13-00455-s001.zip › jpm-2210958-supplementary.pdf]

Supplementary Figure S1. Surveys administered at admission and at discharge to collect information on pa-tient-reported physical and mental health outcomes.

Pre-Exercise Survey

Research Code Number/ID:

Date/Time:

Circle one: UE LE

Circle one: Early ICU DC

Please respond to each item by marking one box per row.

	Never	Almost Never	Sometim es	Often	Almost Always
I had trouble sleeping when I had pain.	☐ 0	☐ 1	☐ 2	☐ 3	☐ 4
I hurt a lot.	☐ 0	☐ 1	☐ 2	☐ 3	☐ 4
I felt unhappy.	☐ 0	☐ 1	☐ 2	☐ 3	☐ 4
I felt weak.	☐ 0	☐ 1	☐ 2	☐ 3	☐ 4
I got tired easily.	☐ 0	☐ 1	☐ 2	☐ 3	☐ 4
I felt sad.	☐ 0	☐ 1	☐ 2	☐ 3	☐ 4

Post-Exercise Survey

Research Code Number/ID:

Date/Time:

Circle one: UE LE

Circle one: Early ICU DC

Please respond to each item by marking one box per row.

During the exercise session. . . . .

	Never	Almost Never	Sometimes	Often	Almost Always
I hurt a lot.	☐ 0	☐ 1	☐ 2	☐ 3	☐ 4
I felt weak.	☐ 0	☐ 1	☐ 2	☐ 3	☐ 4
I got tired easily.	☐ 0	☐ 1	☐ 2	☐ 3	☐ 4
